# Supplementary material for: High-performance nanoscale topological energy transduction
Source: Sci Rep. 2017 Jul 27;7:6736. doi: 10.1038/s41598-017-06965-8 (PMC5532281; doi:10.1038/s41598-017-06965-8)
Supplement: Supplementary file 1 — Supplementary Information [file 41598_2017_6965_MOESM1_ESM.pdf]

High-performance nanoscale topological energy transduction

Timothy M. Philip[1,2,*] and Matthew J. Gilbert[1,2]
[1]Department of Electrical and Computer Engineering, University of Illinois at Urbana-Champaign, Urbana, IL 61801, USA
[2]Micro and Nanotechnology Laboratory, University of Illinois, Urbana, IL 61801, USA
(Dated: May 29, 2017)

Supplementary information includes:

Supplementary Notes

1 Static versus dynamic contributions to inductance . . . 2
2 Influence of island spacing . . . 2

Supplementary Figures

S1 Frequency response calculated using AC NEGF with self-consistency with FDFD and Poisson's equation 2
S2 Schematic of the geometry used to derive current between successive FIs . . . 3

Supplementary Note 1: Static versus dynamic contributions to inductance

We stress the importance of using a fully dynamic electromagnetics solution when calculating inductance as a quasistatic solution by Poisson's equation alone, as the quasistatic solution would fail to capture any flux linkage generated by the circulating currents and adjacent islands. In Fig. S1, we illustrate this importance by recalculating the frequency response of the topological inductor as described in the main text using a self-consistent solution of the AC NEGF equations with Poisson's equation. After self-consistency is reached, the magnetic field is computed from the final current density profile using magnetostatics. We see that over the entire frequency range, self-consistency using Poisson's equation severely underestimates the inductance of the topological inductor by 930%. Most notably, even at low frequencies, where the quasistatic approximation might seem to hold, we still see a dramatic difference between the two electromagnetic solutions. Because Poisson's equation only incorporates charge dynamics, it fails to capture the inductive coupling that is essential to understanding the performance of the topological inductor. Thus the fully electrodynamic method presented here is necessary in regimes where both transient and magnetic dynamics cannot be ignored such as in photoabsorption and magneto-optical Kerr effect spectroscopy.

Supplementary Note 2: Influence of island spacing

To understand the effect of island spacing within the topological inductor, we analytically solve for the wave function of the surface state of a TI between two semi-infinite regions of magnetization as depicted in Fig. S2. The low-energy momentum-space surface state Hamiltonian is given by

$$H = v_F p_x \sigma^y - v_F p_y \sigma^x + V_z \sigma^z, \tag{S1}$$

where p_i are momenta, σ^i are the spin Pauli matrices, and V_z is the Zeeman field. To account for a spatially varying Zeeman field, we Fourier tranform the Hamiltonian into real-space by replacing the momentum p_i with $-i\hbar\partial_i$, giving

$$H = -i\hbar v_F \partial_x \sigma^y + i\hbar v_F \partial_y \sigma^x + V_z(x)\sigma^z, \tag{S2}$$

where $\hbar$ is Planck's constant. Here, the Zeeman field varies in the x direction. We solve for a system as indicated in Fig. S2 where the Zeeman field is a step-wise function of position as

$$V_z(x) = \begin{cases} +M & x < 0 \quad \text{(Region I)} \\ 0 & 0 \leq x < L \quad \text{(Region II)} \\ -M & x > L \quad \text{(Region III)} \end{cases} \tag{S3}$$

Therefore, we can construct an ansatz wave function obtained from simple plane wave solutions for Dirac's equation

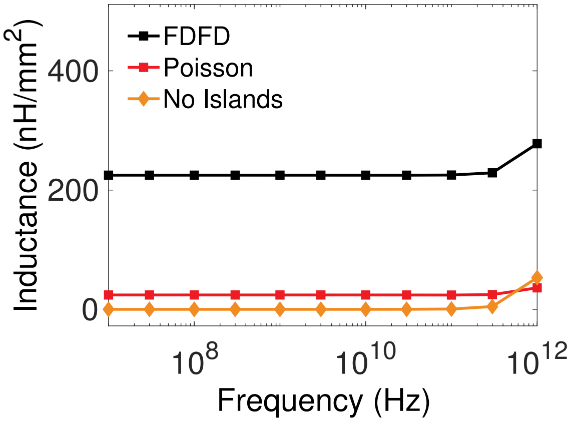

Supplementary Figure S1. Frequency response calculated using AC NEGF with self-consistency with FDFD and Poisson's equation. Because of its ability to capture dynamic magnetic flux linking, FDFD electromagnetic coupling results in an inductance 930% greater than using only Poisson's equation.

for the three regions in Fig. S2. We have set $\hbar = v_f = 1$ for the proceeding, but units will be restored later.

$$\Psi_I(x) = \alpha \begin{pmatrix} 1 \\ \frac{\kappa_x - p_y}{E+V_z} \end{pmatrix} e^{\kappa_x x + i p_y y} \tag{S4a}$$

$$\Psi_{II}(x) = A_1 \begin{pmatrix} 1 \\ \frac{-ip_x - p_y}{E} \end{pmatrix} e^{ip_x x + ip_y y} + A_2 \begin{pmatrix} 1 \\ \frac{ip_x - p_y}{E} \end{pmatrix} e^{-ip_x x + ip_y y} \tag{S4b}$$

$$\Psi_{III}(x) = \beta \begin{pmatrix} 1 \\ \frac{-\kappa_x - p_y}{E+V_z} \end{pmatrix} e^{-\kappa_x x + ip_y y}. \tag{S4c}$$

Since the wave functions within the magnetic domains in regions I and III are exponentially damped and should not vary with the spacing, we focus our analysis on region II. In the DC case, the $\hat{\mathbf{x}}$ momentum is much larger than the $\hat{\mathbf{p}}$ momentum due to the $\hat{\mathbf{x}}$-directed electric field. Therefore by demanding spinor continuity at the interfaces and taking the limit $p_x \gg p_y$, we simplify the wave function to the form

$$\Psi_{II}(x) = \frac{2A_2}{p_x(E+V_z) + iE\kappa_x} \begin{pmatrix} p_x(E+V_z)\cos(p_x x) - 2E\kappa_x \sin(p_x x) \\ \frac{p_x}{E}\left[2E\kappa_x \cos(p_x x) + p_x(E+V_z)\sin(p_x x)\right] \end{pmatrix} \tag{S5}$$

The y-directed current density between the islands can then be found to be proportional to the y-directed velocity given as $\langle v_y \rangle = -v_F \langle \Psi_{II} | \sigma^x | \Psi_{II} \rangle$. After restoring units, we find that

$$\begin{aligned} \langle v_y \rangle = -\frac{4\hbar v_F^2 |A_2|^2}{\kappa_x} \Bigg\{ & \frac{p_x^2(E+V_z)^2}{p_x^2(E+V_z)^2 + (p_y V_z + E\kappa_x)^2} \frac{(\kappa_x - p_y)}{E+V_z} + \frac{\kappa_x}{E p_x^2 (E+V_z)^2 + E^3 \kappa_x^2} \\ & \left[\sin^2(p_x L/\hbar)(p_x(E+V_z) - E\kappa_x)(E(\kappa_x + p_x) + p_x V_z) + E\kappa_x p_x (E+V_z)\sin(2p_x L/\hbar)\right] \\ & + \frac{(p_x(E+V_z)\cos(p_x L/\hbar) - (E\kappa_x + V_z p_y)\sin(p_x L/\hbar))^2}{p_x^2(E+V_z)^2 + (p_y V_z + E\kappa_x)^2} \frac{(-\kappa_x - p_y)}{E+V_z} \Bigg\} \end{aligned} \tag{S6}$$

The current density can then be simply calculated as $J_y = en\langle v_y \rangle$. In this approximation, we find that the velocity and thus current density profile between the magnetic domains varies sinusoidally as a function of position. For our system, $v_F = 151930$ m/s, $M = 0.2$ eV and $E = \mu = 0.1$ eV resulting in an approximation that matches quite well with the peak of the inductance we see with island spacing in the main text.

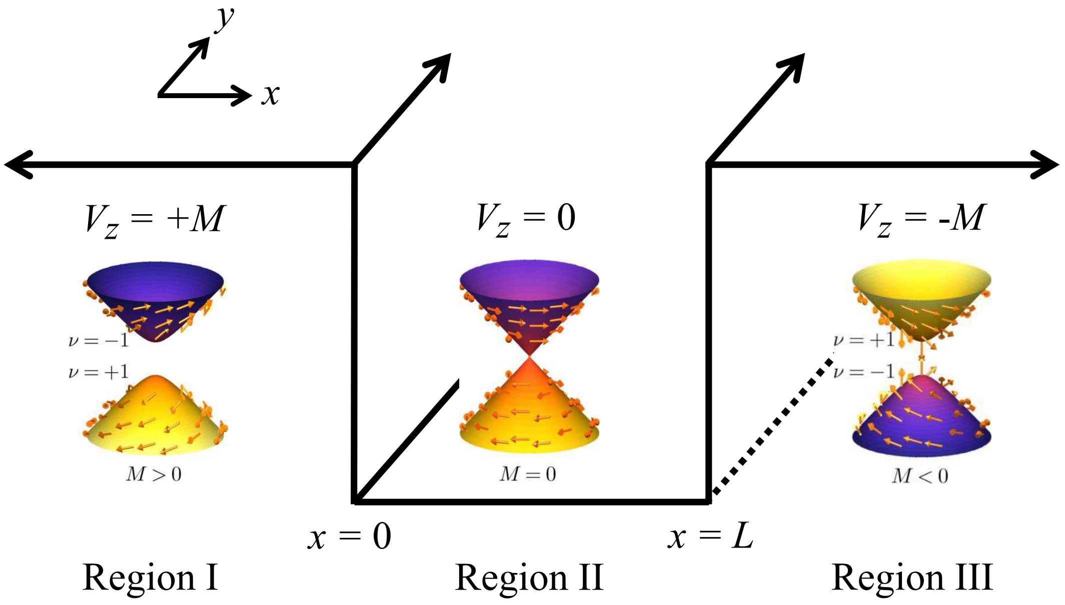

Supplementary Figure S2. Schematic of the geometry used to derive current between successive FIs. The space between the FIs is approximated as a region without magnetization between two semi-infinite regions of $\pm M$ magnetization.
